# Perfluorooctanoic Acid (PFOA) Alters the Structure of the Gut Microbial Community and Colonoid Transcription

**DOI:** 10.3390/cimb48060542

**Published:** 2026-05-22

**Authors:** LinShu Liu, Adrienne B. Narrowe, Jenni Firrman, Karley K. Mahalak, Venkateswari J. Chetty, Johanna M. S. Lemons, Aurélien Baudot, Pieter Van den Abbeele

**Affiliations:** 1 Dairy and Functional Foods Research Unit, Eastern Regional Research Center, Agricultural Research Service, United States Department of Agriculture, Wyndmoor, PA 19038, USA; 2 Cryptobiotix SA, 9052 Ghent, Belgium

**Keywords:** perfluorooctanoic acid, PFOA, gut microbiota, short-chain fatty acids, SCFA, colonoid, transcription

## Abstract

Perfluorooctanoic acid (PFOA) is an environmentally persistent chemical that enters the gastrointestinal tract (GIT) via the food chain, posing a harmful, long-term threat to human health. In response to this challenge, research on the PFOA-GIT interaction is thriving. Currently, studies on the effect of PFOA on the epithelial cells of the GIT and those on its influence on the microbial community are often implemented separately, and less attention has been paid to the combinational effects of the chemical, the gut microbiome and metabolome. In the present study, we co-cultured fecal samples from healthy adults aged 25–70 in the ex vivo SIFR^®^ simulator, adding PFOA at 10 mg/L to represent the accumulated effects of long-term exposure. The results obtained from bacterial cell counting by flow cytometry and shotgun metagenomic sequencing revealed that PFOA was broadly disruptive to the microbiome and that Pseudomonadota emerged as the dominant phylum by replacing Bacteriodota and Bacillota, including key members of short-chain fatty acid-producing groups. Bacterial culture media with and without PFOA were collected and used in human colonoid cell culture for TEER and transcription measurement. It was shown that the PFOA-impacted microbial culture had stronger effects on the cell’s protective functions, in terms of tissue junction tightening, mucin biosynthesis, and immune response, than either untreated bacterial culture or PFOA alone. The results point out the possibility that the combination of PFOA and PFOA-impacted bacterial metabolites more strongly induces a change in epithelial cells’ protective function than either one alone.

## 1. Introduction

Perfluorooctanoic acid (PFOA, C_8_HF_15_O_2_) is a molecule with a hydrophilic carboxylic acid head and a lipophilic fluorinated carbon–carbon backbone. Since its invention together with a broader class of per- and polyfluoroalkyl substances in the late 1930s, and its commercialization beginning in the late 1940s [1], this chemical had been widely used as a key ingredient in consumer and industrial products, such as waterproof or stain-resistant materials, non-stick coatings, and firefighting foams [2,3,4,5,6,7], until 2020, when it was classified as a persistent organic pollutant and banned for production and application globally under the Stockholm Convention on Persistent Organic Pollutants [8]. Despite the discontinuation of its production and use, this chemical persists in the environment, and its disposition remains relevant.

While the inappropriate use of firefighting foams may cause acute symptoms, most of the serious health effects of PFOA were gradually discovered and linked to long-term exposure. PFOA-induced liver damage in animals was observed in 1961, PFOA’s accumulation in human blood was found in 1978, birth defects in lab animals and workers related to PFOA were reported by 1981, and the persistence of PFOA in the environment was confirmed in the 1990s [9,10]. These findings led to regulatory actions and the development of technologies for PFOA removal by novel filtration systems [11,12] and for PFOA detection, such as the use of high-resolution mass spectrometry [13,14]. PFOA is water-soluble, stable in various environmental conditions and resistant to microbial degradation. Eventually, it enters the digestive system via the food chain, which is the main route by which it impacts human health [15,16], despite other exposure routes including through dermal absorption from cosmetics and fabrics [17,18] and by inhalation in certain occupational settings [19,20].

Powered by the achievements in human gut microbiome research, PFOA studies have led to intriguing findings in recent years. Completed studies have shown that PFOA altered the structure of gut microbial communities and affected the level of microbiota-derived metabolites, bile salt conversion, and amino acid biochemical processes [21,22,23,24]. Research on colonic epithelial cell exposure to PFOA has been advanced, and the results have shown complicated influences on tissue junction tightness and barrier integrity, inflammation, and metabolic regulation [25,26]. In current research on PFOA, it is popular to use laboratory animal models and animal epithelial cells or cancer cell lines. One frequently used laboratory animal model is the mouse [27,28]; however, the murine and human gut microbiomes differ in their composition, and while overall, most functions are shared, their expression may vary along with variation in community membership, so the mouse gut can be employed as a model for the human gut with caveats [29,30]. Other models more physiologically relevant to humans are needed to better understand the impact of PFOA on human health matters and to study solutions to mitigate or even diminish the harm of PFOA to human health. Along these lines, another emerging technology is the use of colonoids instead of epithelial cells, which could reveal how this persistent pollutant disrupts intestinal homeostasis at the cellular and molecular levels [21,31]. Furthermore, some results come from direct cell culture with PFOA, which may not be able to paint the whole picture of how PFOA modulates epithelial responses, given the impact of PFOA on the gut microbiome and metabolome; a synergistic effect is possible.

In the present research, we evaluated the impact of PFOA on both human gut microbiota and human colonoids. We first cultivated human fecal samples with PFOA at high dosages to represent long-term exposure and used metagenomic sequencing and SCFA measurements to determine the impact of PFOA on the structure and basic function of the gut microbiome. Bacterial-free supernatants (BFSs) from these cultures were applied to colonoids to evaluate the impact of PFOA-impacted gut microbial metabolomes on colonoid growth and colonoid transcription. 

## 2. Materials and Methods

### 2.1. Materials

Perfluorooctanoic acid (PFOA) and all other chemicals were purchased from MiliporeSigma (Bornerm, Belgium), and colonoids from the distal colon were kindly provided by Prof. G. Wu’s Laboratory of Perelman School of Medicine, University of Pennsylvania (Philadelphia, PA, USA). Fecal samples donated by 18 adults belonging to three age groups of those 25–35, 35–50 and 50–70 years old (*n* = 6 for each) were collected according to the IRB protocol approved by the Ethics Committee of the University Hospital Ghent, Belgium (No. BC-09977).

### 2.2. Ex Vivo Fermentation

Using the ex vivo SIFR^®^ colonic simulation (Cryptobiotix, Ghent, Belgium), the interplay of PFOA and the gut microbiota was evaluated as described previously [32,33]. PFOA was tested at a concentration of 10 mg/L and compared to a no-substrate control (NSC). Gas production was measured as described previously [34]. Individual short-chain fatty acids (SCFAs) were determined on a Gas Chromatograph. Total SCFA and bSCFA amounts were calculated by summing the respective fatty acids. All measurements were triplicated.

### 2.3. Colonoid Treatment and Transepithelial Electrical Resistance (TEER) Measurement

Colonoids were seeded in transwells and cultured to form a confluent monolayer over a week. BFS(TEST) and BFS(NSC) from both the 25–35 and 35–50 age groups, with six donors from each group, were diluted 1:4 in colonoid growth media (pH 7.2–7.4), filter-sterilized through a 0.2 μm filter, and used to treat cells. Experiments were repeated twice. Transepithelial electrical resistance (TEER) readings were measured with an EVOM2 epithelial voltohmmeter and STX4 electrode (World Precision Instruments, Sarasota, FL, USA) [35]. A separate experiment was performed to test whether PFOA in the absence of bacterial metabolites affected barrier function. In this experiment, colonoid monolayers were treated in quadruplicate with 10 mg/L PFOA [Medium(PFOA)] or DMSO (vehicle control) [Medium(Blank)] in colonoid growth media. TEER values were measured over 48 h. Multiple comparisons of a 2-way ANOVA were performed in GraphPad Prism 10 (GraphPad Software, San Diego, CA, USA) to determine significance at different time points.

### 2.4. Bacterial Cell Counts

Bacterial samples were suspended in anaerobic phosphate-buffered saline, stained with 1 μM SYTO 16, and counted for bacterial cell numbers [36] using a BD FACS Verse flow cytometer (BD, Erembodegem, Belgium) [37]. Data were analyzed using FlowJo, version 10.8.1.

### 2.5. DNA Extraction and Sequencing

DNA extraction and metagenomic sequencing were performed by SeqCenter (Pittsburgh, PA, USA). Tagmentation-based sequencing libraries were sequenced on an Illumina NovaSeq X (Illumina, San Diego, CA, USA) to produce 2 × 151 pb paired-end reads.

### 2.6. RNA Extraction and Sequencing

RNA library preparation and sequencing were performed by Azenta Life Sciences (South Plainfield, NJ, USA). RNA extraction was performed using the QIAGEN RNeasy plus and QIAshredder kits (QIAGEN, Santa Clarita, CA, USA). The SMART-Seq v4 Ultra Low Input Kit for Sequencing was used for full-length cDNA synthesis and amplification (Clontech, Mountain View, CA, USA), and the Illumina Nextera XT library preparation kit was used for sequencing library preparation (Illumina, San Diego, CA, USA). Multiplexed sequencing libraries were sequenced on the Illumina NovaSeq using a 2 × 150 bp paired-end (PE) configuration (Illumina, San Diego, CA, USA). Image analysis and base calling were conducted by NovaSeq Control Software (NCS) v. 1.8.1. Raw sequence files were converted to fastq and de-multiplexed using Illumina bcl2fastq 2.20 software. One mismatch was allowed for index sequence identification.

### 2.7. Bioinformatics and Statistical Analysis

Raw shotgun metagenomic sequencing data was preprocessed using BBDuk v. 39.01 [38]. Trimmed, filtered reads were used as input to MetaPhlAn v. 4.1.1 with the mpa_vJun23_CHOCOPhlAnSGB_202403 reference database [39]. Read-based functional profiling was performed using HUMAnN v. 4.0.0 [40].

Testing for significant differences in taxon abundance with treatment was performed using MaAsLin3 [41] with the relative abundance table and flow cytometry cell counts for abundance normalization. Multiple testing correction was performed using the Benjamini–Hochberg method.

RNA-Seq data was quality-trimmed using BBDuk as above. Transcript quantification was performed using kallisto v. 0.48.0 [42] using a reference prepared from the Ensembl human genome release 113 (Homo_sapiens.GRCh38.113). Kallisto-estimated abundances were used as input to DESeq2 v. 1.44.0 [43] to calculate differential gene expression using Wald’s test. Lists of differentially expressed genes were used as input to enrichR [44] for GO bioprocess enrichment analysis. Other statistical analyses and visualizations used R/RStudio (v.4.1.3) using the packages: tidyverse (v.1.3.1) [45], vegan (v.2.6-2) [46], ape (v.5.6-2) [47], and ggvolc [48]. TEER plots were also created using GraphPad Prism 10 (GraphPad Software, San Diego, CA, USA).

Further details on methods and materials can be found in the Supplementary Materials.

## 3. Results

### 3.1. PFOA Induced Changes to Microbial Community

**Figure 1:**
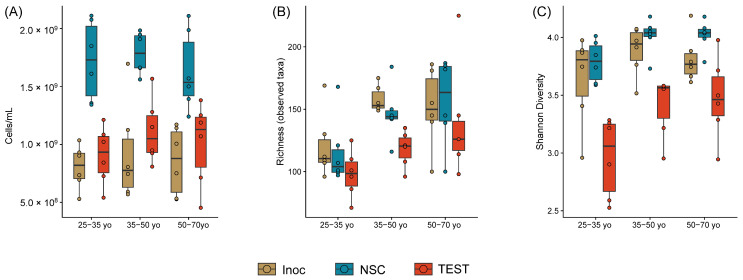
Microbial community growth and diversity is inhibited by PFOA addition. (**A**) Cell counts from flow cytometry. (**B**) Richness as measured by the number of species identified. (**C**) The Shannon Diversity Index. Boxplots show the median and IQR with points overlaid to represent six individual donors per age group.

To assess the effects of PFOA on microbial communities, we first measured bacterial cell counts and combined this with the shotgun metagenomic sequencing of the original fecal samples (Inoc), the cultures with added PFOA (TEST), and those without PFOA (NSC). As shown in Figure 1A, gut microbiota grew significantly in the SFIR^®^ bioreactor in both the NSC and TEST samples compared to the inoculum in all age groups over the 48 h incubation period (q < 0.05) with the single exception of the 35–50 age group, where the TEST samples did not significantly exceed inoculum cell counts. This growth was further impacted by the addition of PFOA as all the NSC samples had significantly higher cell counts than the TEST samples (q < 0.01). To consider how the treatments changed the within-sample community composition, we used the metagenomic community profile. The analysis of intra-sample richness (numbers of unique species) (Figure 1B) showed significant changes only for the 35–50 age group, for which the values were reduced by 48 h incubation (q = 0.002) and further reduced by PFOA exposure (q = 0.004). Considering evenness as well as richness, the Shannon Diversity Index nearly reversed the trend for richness. Here, the index of all comparisons differed significantly, with only the Inoc/NSC comparison for the 25–35 and 35–50 age groups not differing significantly (q < 0.05). In all cases, the PFOA-treated samples had significantly lower intra-sample diversity than the inoculum and the untreated controls (q < 0.005), while the untreated controls equaled or exceeded the inoculum in species richness and diversity (Figure 1C).

**Figure 2:**
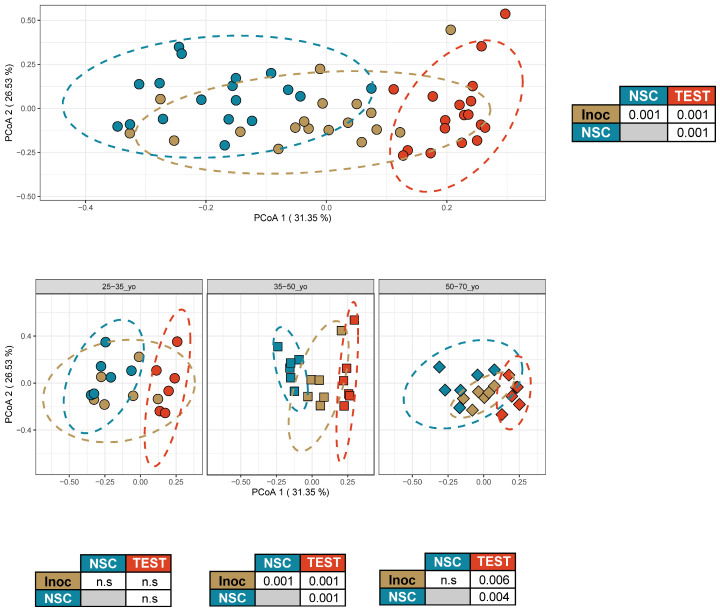
A principal coordinate analysis of weighted UniFrac distances with all samples plotted together regardless of age (**top**) and separated by age (**bottom**). Point positions are the same in the top and bottom plots. Tables indicate *p*-values from PERMANOVA testing for the significant clustering of points by treatment group. Dashed lines represent 95% confidence intervals. Brown, Inoculum; Blue, no-substrate control (NSC); Red, TEST (PFOA treated).

Comparing samples at the whole community level, PCoA using the weighted UniFrac distance showed that the samples clustered by treatment when all ages were considered together (*p* = 0.001; Figure 2, top panel). The influence of PFOA was also compared by age groups separately (Figure 2, bottom panels). For the 25–35 year age group, there was overlap between the Inoc and NSC samples (p: ns) and between the Inoc with TEST samples (p: ns); for the 35–50 age group, the samples clustered by treatment (*p* = 0.001); for the 50–70 age group, the TEST and NSC samples (*p* = 0.004) and the TEST and Inoc samples (*p* = 0.006) differed significantly in overall community structure, while the inoculum and controls samples did not differ.

**Figure 3:**
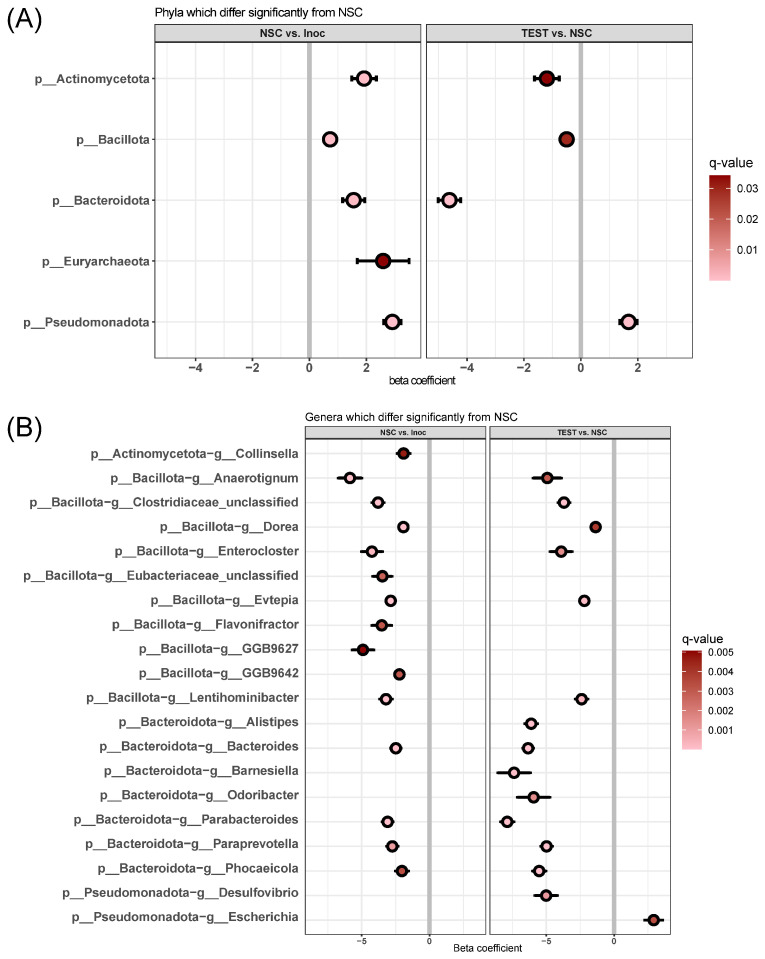
The results of the MaAsLin3 test for differential abundance by (**A**) phylum level and (**B**) genus level. The x-axis shows the beta coefficient for the comparison indicated at the top of the plot. The significance of abundance change (q-value) is shown by point color. Only the top 20 genera with the largest changes are shown.

The alpha- and beta-diversity analyses reveal that sharp variations occurred with the gut microbial communities during the in vitro culture and specifically with PFOA exposure. Examining the changes in community membership and abundance that drove these observed differences showed that among the top five phyla detected in the three age groups, Bacillota and Bacteroidota are two dominant phyla in the inoculum and NSC samples; however, PFOA treatment nearly eliminated Bacteroidota, which decreased significantly in the TEST samples as compared to NSC (29–37% in NSC vs. 2–6% in TEST, q < 0.0001) (Supplementary Table S1, Supplementary Figure S1). Similarly, but less drastically, Actinomycetota and Bacillota decreased in the TEST vs. NSC samples; while the phylum Pseudomonadota (former name, Proteobacteria) increased significantly from <2% relative abundance (RA) in Inoc and <5% in NSC to 30.55% for the 25–35 age group and 15% for the other two groups (Supplementary Table S1). The results of taxonomic differential abundance analysis indicate that PFOA exposure decreased the abundance of TEST samples relative to NSC for all phyla except Pseudomonadota (beta coefficient, 1.67), which was comparable to samples without PFOA (beta coefficient, 2.91) (Figure 3A).

Next, we analyzed the compositional changes within the microbial communities at the genus level. The top 20 genera that changed significantly in abundance with exposure to PFOA or with time in NSC relative to the inoculum are listed in Figure 3B. Among them, the abundance of 18 increased significantly in the NSC incubations in the absence of PFOA, except for the genus from sulfate-reducing bacteria (SRB), *Desulfovibrio*, and the species from genus *Escherichia*. With the addition of PFOA to the system, the growth of all these genera was suppressed, except for the genus *Escherichia*, which was able to grow positively and significantly. In a comparison of bacteria belonging to phylum Bacillota, genus SRB and genera from phylum Bacteroidota were further suppressed. Other genera that were commonly recognized as SCFA producer/consumer bacteria are listed in Supplementary Figure S2. The genera Anaerostipes and Eubacterium were present at low abundances, which were followed by *Prevotella*. Interestingly, *Ruminococcus* and *Roseburia* from some donors were promoted by PFOA, while the growth of *Akkermansia muciniphila*, *Faecalibacterium prausnitzii* and *Blautia* was suppressed.

### 3.2. PFOA Altered Gut Microbial Metabolism

**Figure 4:**
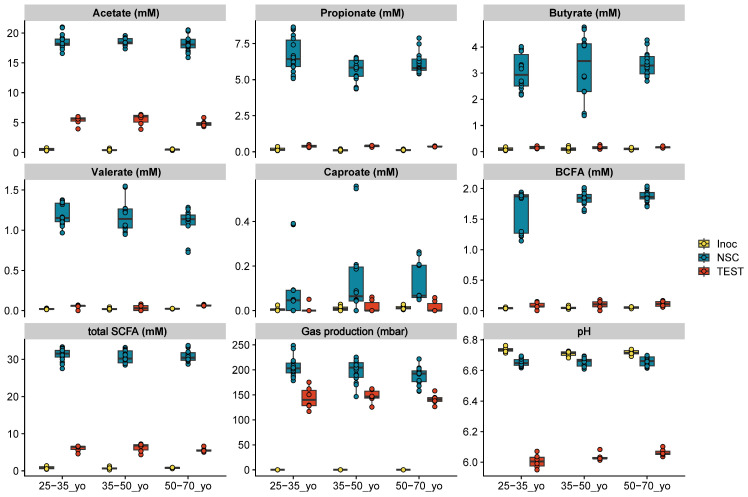
Concentrations of short-chain fatty acids (SCFAs) and gas as measures of fermentation.

As for the effect of PFOA on gut microbial metabolism in the in vitro culture, SCFA, gas production, and pH were measured as indicators of microbial fermentation activity. As shown in Figure 4, all SCFAs and gas production increased significantly over 48 h in the measured NSC samples. However, the increase in SCFAs, while significantly higher than in the inoculum samples, was suppressed by PFOA inclusion (q < 0.05) with the exception of caproate and BCFAs. These increases over baseline were overshadowed by the increases in the untreated samples, and in all cases, the untreated samples significantly outproduced the PFOA-treated samples (q < 0.01; Figure 4). The 48 h incubation in the presence of PFOA also reduced the medium pH on average by 0.7 units (6.72–6.03), while the incubation conducted in the absence of PFOA had a negligible effect on pH change (6.66–6.60) (Figure 4). These results suggest that the presence of PFOA may induce bacterial metabolism that is different from that in its absence. At the end of fermentation, the BFSs from two types of cultures, BFS(TEST) and BFS(NSA), were isolated and evaluated for cellular activities on colonoid cells.

### 3.3. PFOA Reduced TEER Values

**Figure 5:**
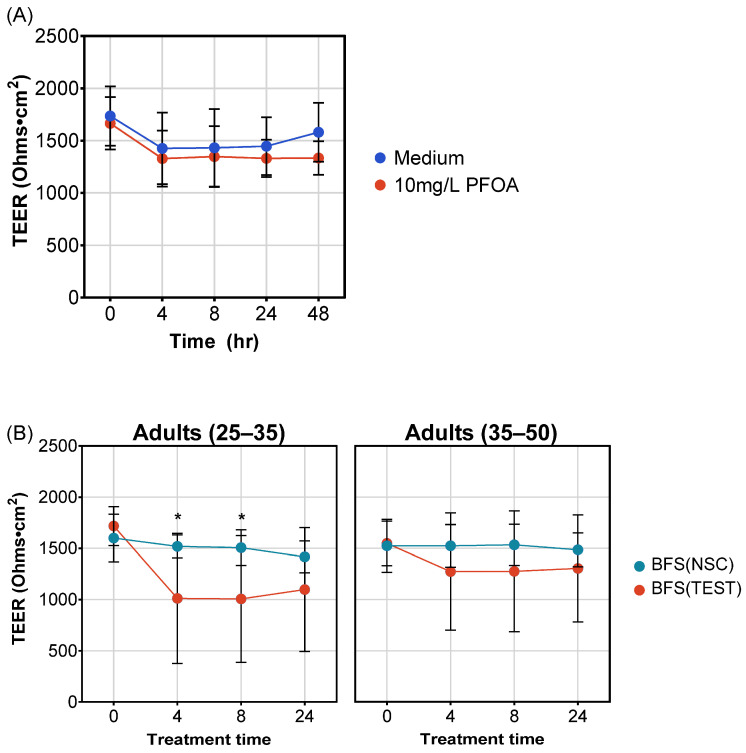
Transepithelial electrical resistance measurements over time for colonoid monolayers (**A**) treated with 10 mg/L PFOA or vehicle control; (**B**) treated with BFS from NSC and PFOA-treated bacterial cultures isolated from the 25–35 (**left**) and 35–50 (**right**) age groups of adults (* indicates adj. *p*-value < 0.05 for that time point).

Figure 5A shows the barrier strength of 2D colonoid monolayers grown on transwells and treated with media containing either vehicle, PFOA, or supernatant from gut microbial cultures treated with NSC or PFOA. Monolayers treated with Medium(PFOA) did not have a significant impact on barrier function compared to Medium(Blank), the vehicle control. BFS(TEST) supernatants from the 25–35 age group caused a statistically significant reduction in the barrier function of the monolayer at 4 and 8 h of treatment compared to BFC(NSC) (Figure 5B). Barrier function was not significantly different at any time point for the 35–50 age group. The results of these two experiments suggest that although PFOA on its own showed no significant effect on the barrier function of the epithelial cell layer, gut bacteria treated with PFOA may produce metabolites that weaken it.

### 3.4. Transcription of Colonoids Incubated with BFS

**Figure 6:**
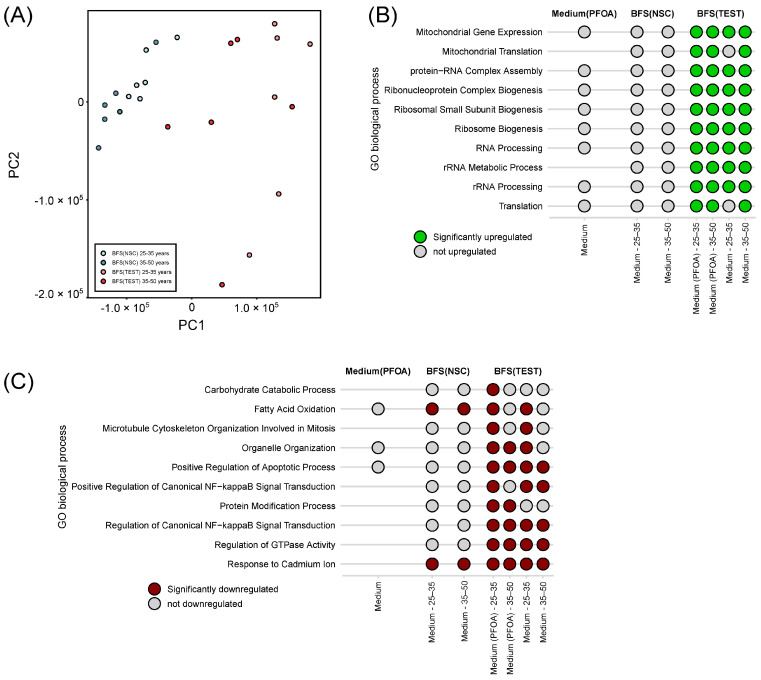
(**A**) Principal component analysis (PCA) of transcript levels for BFS-treated colonoids. (**B**) Upregulated Gene Ontology (GO) bioprocesses. (**C**) Downregulated Gene Ontology (GO) bioprocesses.

RNA was extracted from the colonoids collected from the TEER experiments to determine the transcriptional activity of the cells driven by PFOA or bacterial metabolites, either separately or in combination. The principal coordinate analysis of the global transcriptional levels of the colonoids treated with BFS(TEST) and BFS(NSC) showed a clear separation of samples by treatment type, with limited effects seen associated with age group (Figure 6A). Furthermore, samples treated with BFS(NSC) clustered together and showed less variability in the transcriptome compared to those treated with BFS(TEST), which were more broadly distributed. This clearly indicates that there is a difference in transcription overall by BFS and a little difference with respect to age. To further explore the gene–treatment pattern, the differentially expressed genes (DEGs) identified by DESeq2 were used for pairwise comparisons under four distinct conditions: “Medium(PFOA) vs. Medium(Blank)”, “BFS(NSC) vs. Medium(Blank)”, “BFS(TEST) vs. Medium(PFOA)”, and “BFS(TEST) vs. Medium(Blank)”. The four comparisons provided information on the effects of PFOA, bacterial metabolites, PFOA-impacted bacterial metabolites, and the effects of the mixture of PFOA and PFOA-impacted bacterial metabolites, respectively. The results of the different treatments can be visualized as a volcano plot (Supplementary Figure S3). For the 25–35 age group, 701 DEGs were upregulated and 67 were downregulated for “Medium (PFOA) vs. Medium (Blank)”, 1759 DEGs were significantly upregulated and 1191 were downregulated for “BFS(NSC) vs. Medium (Blank)”, 2527 DEGs were upregulated and 2379 were downregulated for “BFS(TEST) vs. Medium (PFOA)”, and 2433 DEGs were significantly upregulated and 1859 were downregulated for “BFS(TEST) vs. Medium(Blank)”. All changes in regulation were statistically significant. The results indicate that PFOA-impacted bacterial metabolites have a stronger influence on colonoid gene expression than the metabolites produced in the absence of PFOA. Similar effects of PFOA-impacted and bacterial cultural metabolites on the 35–50 age group were also seen (Supplementary Figure S3).

Pathway enrichment analysis was performed using enrichR to identify Gene Ontology (GO) bioprocesses that were upregulated or downregulated in response to the treatment (Supplementary Materials). The top 10 upregulated and top 10 downregulated bioprocesses are listed in Figure 6B,C, using the BFS(TEST) vs. Medium(PFOA) results of the 25–35 age group as the reference for selection. Considering the DEG comparisons as described above, these bioprocesses were upregulated only when treated with BFS(TEST), a mixture of PFOA and PFOA-impacted bacterial metabolites, and not in the presence of either of them separately for these top 10 GO bioprocesses. Similarly, except for “response to cadmium ion” and “fatty acid oxidation”, the downregulation of genes suggests an additive effect of bacterial metabolites and PFOA. When the colonoids were treated with BFS(TEST), the consistent upregulation of GO bioprocesses associated with transcription and translation (ribosome assembly) was observed with limited exceptions (Figure 6B). In contrast, for processes that were downregulated by BFS(TEST), the response of the bioprocess of colonoids showed a more variable, GO-dependent pattern (Figure 6C).

Next, we examined transcriptomic data in the context of specific functional properties. For DEGs encoding tissue junction tightening (Table 1), the addition of PFOA alone to the medium had no significant effect on the five genes. The treatment with bacterial metabolites downregulated gene *Claudin-3* (*CLDN3*) in both age groups and gene *TFF3* in the 25–35 age group. The treatment of PFOA-impacted bacterial metabolites results in the downregulation of *Tight Junction Protein 1* (*TJP1*), *Occludin* (*OCLN*), and *CLDN3* in both age groups; this was extended to gene *TFF3* as PFOA was present with the PFOA-impacted bacterial metabolites. The gene *DNMT3* in both age groups was upregulated by the treatment with PFOA-impacted metabolites, or its mixture with PFOA, and for the 25–35 age group by bacterial metabolites.

Table 2 shows the effect of PFOA and metabolites on mucin biosynthesis. In total, the transcription of 20 genes associated with intestinal mucin synthesis and accumulation was detected in this experiment (Table 2). Among these 20 genes, there was a variable response to the test conditions. Five genes were significantly promoted by some treatments: Gene *Nuclear factor (NF)-kappa-B1* (*NFKB1*) was upregulated by PFOA; *NFKB1* in both age groups was also upregulated by bacterial metabolites and the mixture of PFOA and PFOA-impacted bacterial metabolites. The genes *MUC13* and *MUC17* in the young group were upregulated by the treatment of PFOA-impacted bacterial metabolites and its mixture with PFOA; gene *MUCL3* in the young group treated with the mixture of PFOA and PFOA-impacted bacterial metabolites, as well as gene *NFKBIL1* in both age groups treated with PFOA-induced bacterial metabolites, was upregulated. The other 15 genes were significantly downregulated, mainly by the treatment of PFOA-impacted metabolites and the mixture with PFOA.

Another critical element of the GIT’s protective system is immune activity. Again, PFOA alone showed no effect on the four genes activated by the other three treatments in the present experiment. The three treatments significantly upregulated the gene expression of *Toll-like receptor 4* (*TLR4*) and downregulated gene *LY2* in the two age groups. Gene *TLR6* in both age groups was upregulated by either bacterial metabolites or PFOA-induced bacterial metabolites. Gene *TLR3* in the 35–50 age group was downregulated by the treatment with the mixture of PFOA and PFOA-impacted bacterial metabolites. Toll-like receptors are key sensors of immune system. Upregulating *TLR* genes suggests the activation of the immune system in response to the BFS. Table 3 reveals that the PFOA-impacted metabolites or their combination with PFOA strongly influenced the transcription of colonoids, while PFOA alone had much less strong effects under the present experimental conditions.

## 4. Discussion

In most cases, PFOA exerts its health effects in a gradual and long-lasting manner. To account for the potential effects of PFOA within a benchtop experimental model employed over a short time period, we used a dosage of 10 mg/L PFOA in the SIFR^®^ colonic stimulation, which is 10 times higher than that in the Applicability Determination Index issued by the U.S. EPA [49]. Based on the results shown in Figure 1, all in vitro gut microbial communities demonstrated high proliferative activity under standard culture conditions in the control samples, while the environmental stress of exposure to PFOA suppressed bacterial growth and led to the loss of ecological diversity, as evidenced by a significant reduction in the Shannon Index. Three healthy adult age groups were tested, representing younger (25–35 years), midlife (35–50 years) and older (50–70 years) adults. The 35–50 age group was the only group that significantly declined in species richness over time with PFOA treatment, indicating a shift in subset taxa to more resilient ones and the more vulnerable to present environmental conditions and suggesting greater vulnerability to the effects of PFOA in the gut microbiomes of donors of this age group than the other two groups. Despite this decrease, the diversity of the 35–50 age group remained stable (Figure 1C), indicating that the remaining species were relatively evenly distributed. In contrast, both the species richness and Shannon Index for the 25–35 age group did not change significantly during 48 h of incubation under standard conditions, exhibiting community structural integrity in untreated samples. The characteristic difference in the ecological structure between the 25–35 and 35–50 age groups was confirmed by beta-diversity analysis (Figure 2), which revealed an overlap between the Inoc and NSC samples (p: ns) for the 25–35 age group and divergence for the 35–50 age group (*p* = 0.001). Due to the apparent difference in the response of the two age groups to PFOA-impacted perturbation, the BFS from these two age groups was later used to compare the effects of a PFOA-challenged microbiome on colonoid cell culture.

PFOA exposure significantly altered the gut microbial community even at the phylum level (Supplementary Figure S1 and Supplementary Table S1). In PFOA-exposed samples, Pseudomonadota became a dominant phylum at the cost of Bacteroidota and Bacillota. For in vivo studies, the ability of Pseudomonadota to outcompete other taxa was observed and attributed to several factors, including PFOA-induced “leaky gut”, which increases intestinal permeability, resulting in oxygen-rich inflammatory niches that favor facultative microbial components like members of Pseudomonadota, which disrupted the metabolism of bile salts and induced oxidative stress and redox shift [50,51,52,53]. For the present experiment conducted with SIFR^®^ technology, we linked this to the differences in PFOA sensitivity among Pseudomonadota and other bacterial phyla. Pseudomonadota possess a different cell membrane structure from phyla Bacteroidota and Bacillota that is more resistant to surfactant-like chemicals [54,55]; Pseudomonadota are known to be rich in genes associated with antibiotic and chemical resistance, which may provide an adaptive advantage upon exposure to PFOA [56,57]; furthermore, members of this phylum are capable of effectively forming biofilms, which may protect bacterial cells from PFOA infiltration [57,58,59]. These features may favor Pseudomonadota and like bacteria by taking the niches left by Bacillota and Bacteroidota and thriving. 

The loss of Bacillota and Bacteroidota can have important implications for the production of SCFAs, which are markers of gut microbiome function. Reflected in the shift in the microbial composition from Bacillota/Bacteroidota domination to another major phylum Pseudomonadota, genus-level reductions in SCFA-producing or -consuming taxa were detected. The fraction of Bacillota and Bacteroidota members, such as members of Lachnospiraceae, Oscillospiraceae, Eubacteriales, and g. *Bifidobacterium*, was reduced (Figure 3B and Figure S2); subsequently, SCFA levels dropped across the three age groups, especially for acetate, propionate, and butyrate (Figure 4). These three major SCFAs play a powerful role not only as both metabolic fuel and signaling molecules but also in shaping the protective functions of the GIT. From the experimental results obtained from laboratory animal models, the reduction in SCFAs weakens the tight junction of colonic epithelial tissues and thins the mucus layer that promotes vulnerability to leaky gut and pathogen penetration, and this may also elevate autoimmunity and pro-inflammatory conditions [60,61,62,63,64,65]. Besides the decrease in SCFA production and the measured gas reduction, the pH drop may also indicate the PFOA-impacted change in bacterial metabolites (Figure 4). The pH dropped from 6.72/6.66 (Inoc) to 6.60 (NSC) and 6.03 (TEST), although this is still in the range of healthy colonic conditions. This drop could be due to PFOA’s acid nature that thinned the buffered function of inorganic salts used in the media, which may also indicate the production of acid metabolites that are not found in the NSC sample and need to be identified in coming experiments.

To consider the effects of PFOA or gut microbial metabolites on the integrity of the GIT epithelial barrier, we applied these substrates to colonoids. The results from TEER measurement showed that PFOA alone, under the present experimental conditions, had little effect on junction tightness, but the PFOA-impacted BFS lowered the TEER values, indicating that the combination of microbial metabolites with PFOA or microbial metabolites following PFOA challenge may have an important effect on weakening the gut epithelium (Figure 5). This finding was also seen in the transcriptomic data from the colonoids treated with these substrates. As shown in Table 1, treatment with PFOA-impacted bacterial metabolites or its combination with PFOA dramatically downregulated the genes encoding for colon tissue junction tightening, suggesting the loosening of cell–cell adhesion and an increase in permeability to luminal contents that may enhance susceptibility to inflammation or immune activation [66,67]. This was supported by GO bioprocess analysis (Supplementary Materials), where GO bioprocesses for epithelial cell differentiation (GO:0030855) and Tight Junction Assembly (GO:0120192) were significantly downregulated in the BFS(TEST) vs. Medium(Blank) comparison for the 35–50 age group, while only epithelial cell differentiation (GO:0030855) was significantly downregulated in the 25–35 age group. Similarly, Cell–Cell Junction Maintenance (GO:0045217) and Adherens Junction Maintenance (GO:0034334) were downregulated in the 35–50 age group in the TEST vs. PFOA alone comparison. These results support the idea that there is an additive effect of PFOA and microbial metabolism, which may negatively impact GIT epithelial integrity. The downregulation of genes encoding mucin biosynthesis (Table 2) suggests the thinning or dysfunction of the mucin barrier, which increased colonic permeability and correlates with inflammatory bowel disease [68,69]. The upregulation of genes for immune response (Table 3) indicates that incubation with PFOA triggered a pro-inflammatory or immunomodulatory response. This could be considered the consequence of combinational effects, directly from PFOA, including the loosening of junction tightening and weakened mucin dynamics [70,71].

Colon-protective functions integrate three elements, the epithelial tissue barrier, mucin biosynthesis, and active immune response, contributing to gut microbial homeostasis. In the present research, we explored how PFOA disrupted the gut microbial structure and composition and, consequently, how the metabolites produced and PFOA together alter the three basic functions of the gut-protective system by up- or downregulating responsible genes. The present study did not identify the untargeted metabolites produced in PFOA-impacted gut microbial culture, nor did it touch on the connection of the three protective functions, leaving these topics for subsequent research.

## 5. Conclusions

The exposure of an ex vivo gut microbial community to PFOA reshaped the community structure by suppressing commensal bacterial growth, including that of SCFA-producing and -consuming taxa; in turn, Pseudomonadota became a dominating phylum. As such, the altered metabolite profile impacted colonoid cells’ transcription and barrier integrity response, and the transcriptional mutagenesis of colonoids may point to an unseen peril of the persistent chemical on human health. Ongoing work will continue research on the coupling of the PFOA-impacted gut microbiome with the transcriptional mutagenesis of intestinal epithelial cells.

## Figures and Tables

**Table 1:** DEGs encoding for junction tightening of colonoids.

Gene ID	Annotation	Pair One	Pair Two		Pair Three		Pair Four	
			25–35 Years	35–50 Years	25–35 Years	35–50 Years	25–35 Years	35–50 Years
OCLN	Occludin	-	-	-	−0.721	−0.703	−0.704	−0.757
TJP1	Tight Junction Protein 1	-	-	-	−0.480	−0.701	−0.594	−0.815
CLDN3	Claudin-3	-	−0.334	−0.330	−0.380	−0.606	−0.364	−0.590
TFF3	Trefoil Factor Family 3	-	−0.331	-	-	-	−0.460	−0.401
DNMT3A	DNA Methyl-transferase 3A	-	0.562	-	0.424	0.386	0.626	0.588

Pair One, medium (PFOA) vs. medium (none). Pair Two, BFS(NSC) vs. medium (none). Pair Three, BFS(TEST) vs. medium (PFOA). Pair Four, BFS(TEST) vs. medium (none).

**Table 2:** DEGs encoding for mucin synthesis of colonoids.

Gene ID	Annotation	Pair One	Pair Two		Pair Three		Pair Four	
			25–35 Years	35–50 Years	25–35 Years	35–50 Years	25–35 Years	35–50 Years
MUC1	Mucin 1	-	-	-	-	−0.942	-	−0.949
MUC12		-	-	-	−0.452	−0.527	-	−0.601
MUC13		-	-	-	0.635	-	0.610	-
MUC17		-	-	-	0.763	-	0.990	-
MUC2		-	-	-	-	−3.000	-	−3.415
MUC20		-	-	-	−1.079	−0.901	−0.877	−0.698
MUC20-OT1	MUC20 overlapping transcript	-	-	-	−1.387	−1.081	−1.053	−0.746
MUC3A		-	-	-	-	-	-	-
MUC4		-	-	-	−0.710	−0.772	-	-
MUC5AC		-	-	-	-	-	-	-
MUC5B		-	-	-	-	-	-	-
MUCL3		-	-	-	-	-	2.957	-
TFF3	Trefoil factor family 3	-	−0.331	-	-	-	−0.460	−0.401
PPARα	Peroxisome proliferator-activated receptor alpha	-	-	-	-	-	-	-
FUT2	Fucosyltransferase 2	-	-	−1.011	-	-	−0.659	−0.714
NFKB2	Nuclear factor (NF)-kappa-B2	-	-	-	-	−0.696	-	-
NFKBIA		-	−0.601	-	−0.326	−0.476	−0.405	−0.555
NFKBIZ		-	−1.62	-	−1.857	−1.434	−1.159	−1.165
NFKBIL1		-	-	-	0.427	0.393	-	-
NFKB1		1.398	1.471	1.301	-	-	1.565	1.297

Pair One, medium (PFOA) vs. medium (none). Pair Two, BFS(NSC) vs. medium (none). Pair Three, BFS(TEST) vs. medium (PFOA). Pair Four, BFS(TEST) vs. medium (none).

**Table 3:** DEGs encoding for immune activity of colonoids.

Gene ID	Annotation	Pair One	Pair Two		Pair Three		Pair Four	
			25–35 Years	35–50 Years	25–35 Years	35–50 Years	25–35 Years	35–50 Years
TLR4	Toll-like receptor 4	-	0.476	0.393	1.059	1.083	0.892	0.916
TLR3		-	-	-	-	−0.431	-	-
TLR6		-	5.871	6.776	-	-	6.031	5.718
LYZ	Lysozyme	-	−0.392	−0.459	−0.527	−0.389	−0.644	−0.505

Pair One, medium (PFOA) vs. medium (none). Pair Two, BFS(NSC) vs. medium (none). Pair Three, BFS(TEST) vs. medium (PFOA). Pair Four, BFS(TEST) vs. medium (none).

## Data Availability

Raw sequencing reads (DNA and RNA) will be made available in the NCBI Sequence Read Archive under accession number PRJNA1456031.

## Supplementary Materials

The following supporting information can be downloaded at https://www.mdpi.com/article/10.3390/cimb48060542/s1.
