# Supplementary material for: Perfluorooctanoic Acid (PFOA) Alters the Structure of the Gut Microbial Community and Colonoid Transcription
Source: Curr Issues Mol Biol. 2026 May 22;48(6):542. doi: 10.3390/cimb48060542 (PMC13297463; doi:10.3390/cimb48060542)
Supplement: Supplementary file 1 [file cimb-48-00542-s001.zip › cimb-4310344-supplementary.pdf]

SUPPLEMENTARY INFORMATION:

Perfluorooctanoic acid (PFOA) alters the structure of the gut microbial community and colonoid transcription

LinShu Liu[1*], Adrienne B. Narrowe[1], Jenni Firrman[1], Karley K. Mahalak[1], Venkateswari J. Chetty[1], Johanna M. S. Lemons[1], Aurélian Baudot[2], Pieter Van den Abbeele[2]

1 - Dairy and Functional Foods Research Unit, Eastern Regional Research Center, Agricultural Research Service, United States Department of Agriculture, Wyndmoor, PA 19038, USA

2 - Cryptobiotix SA., Ghent 9052, Belgium

*Corresponding Author: LinShu Liu, linshu.liu@usda.gov +1-215-233-6486

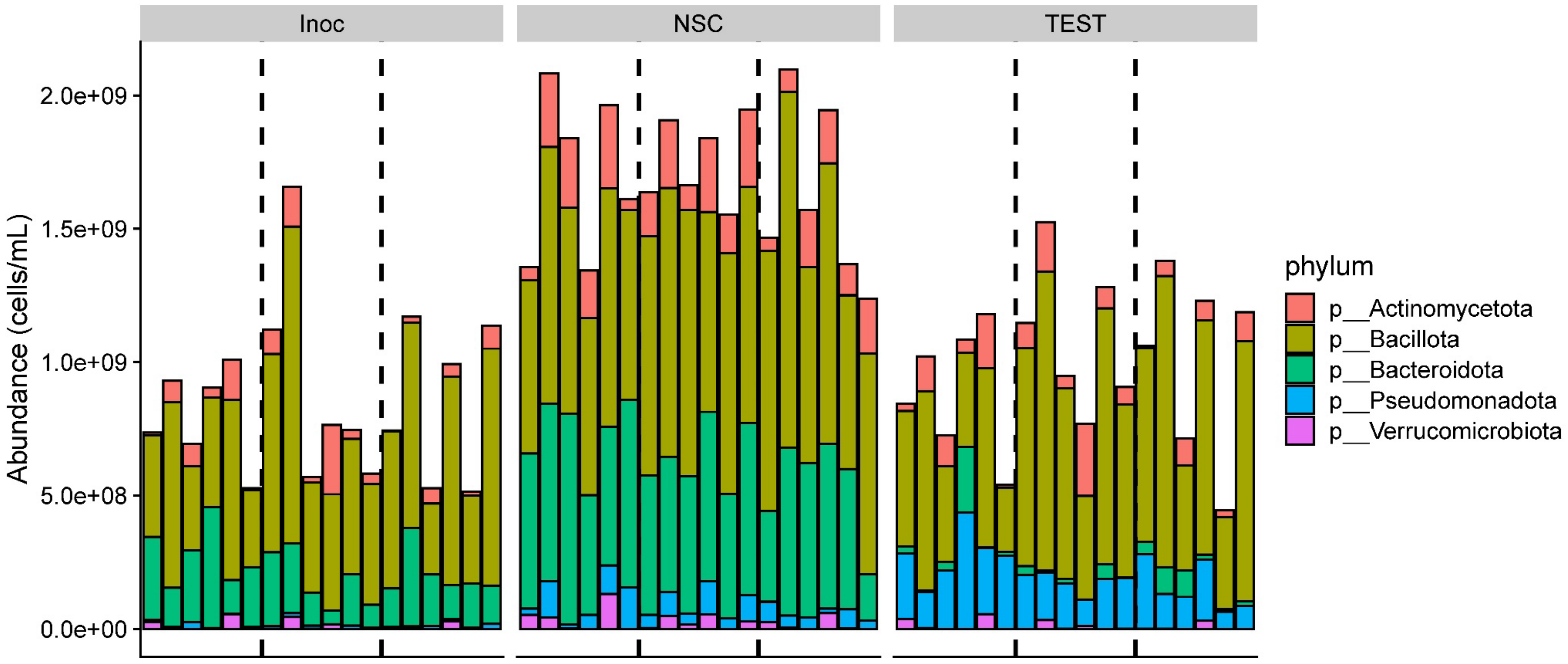

Figure S1 – Phylum level abundances in the three tested conditions. Each bar represents the microbial community of one donor. Dashed vertical lines separate the age groups.

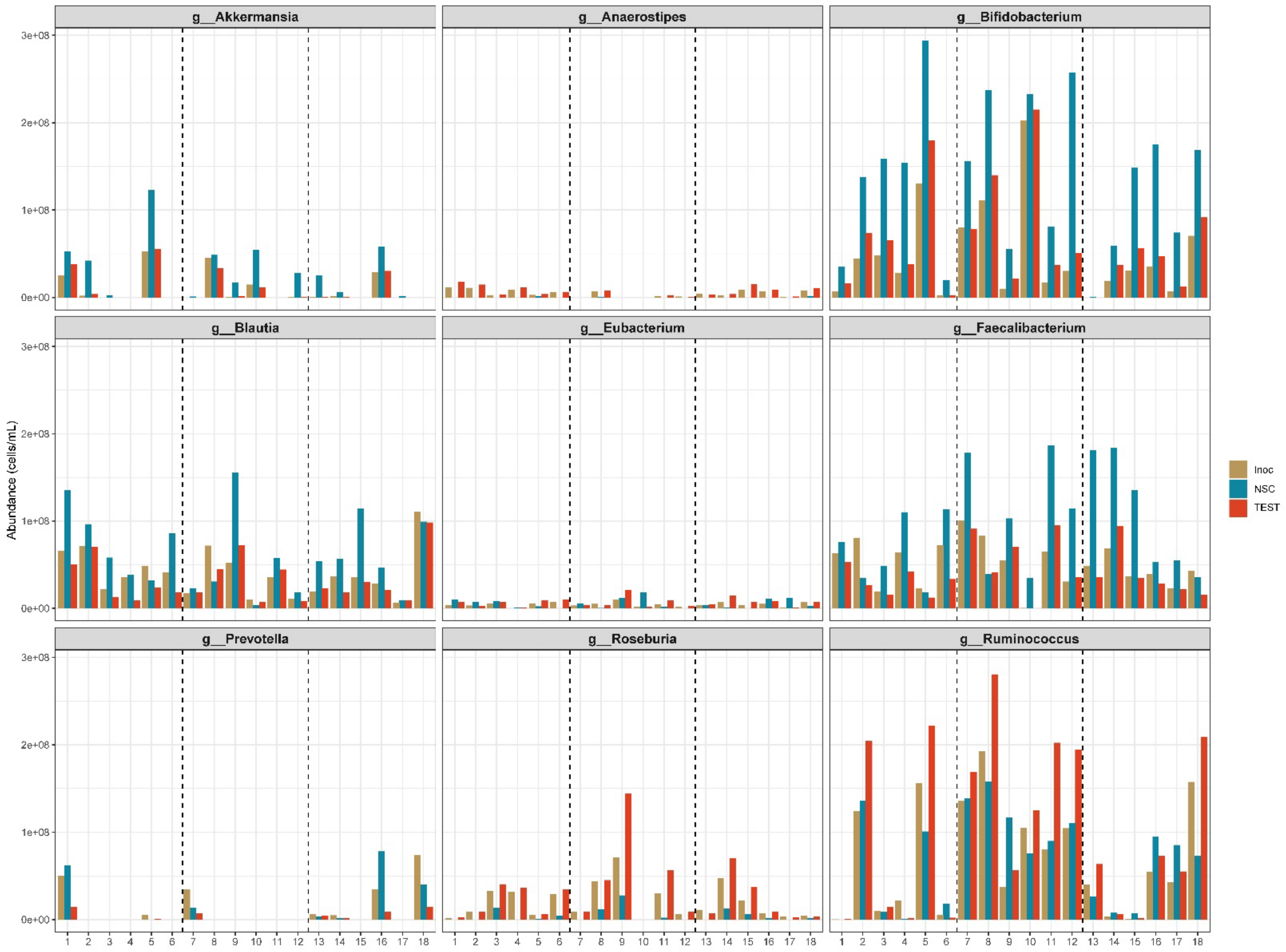

Figure S2 – Abundances of selected genera in the three tested conditions. Each bar represents the microbial community of one donor. Dashed vertical lines separate the age groups. Colors indicate Inoculum (gold), NSC (blue), and PFOA (red).

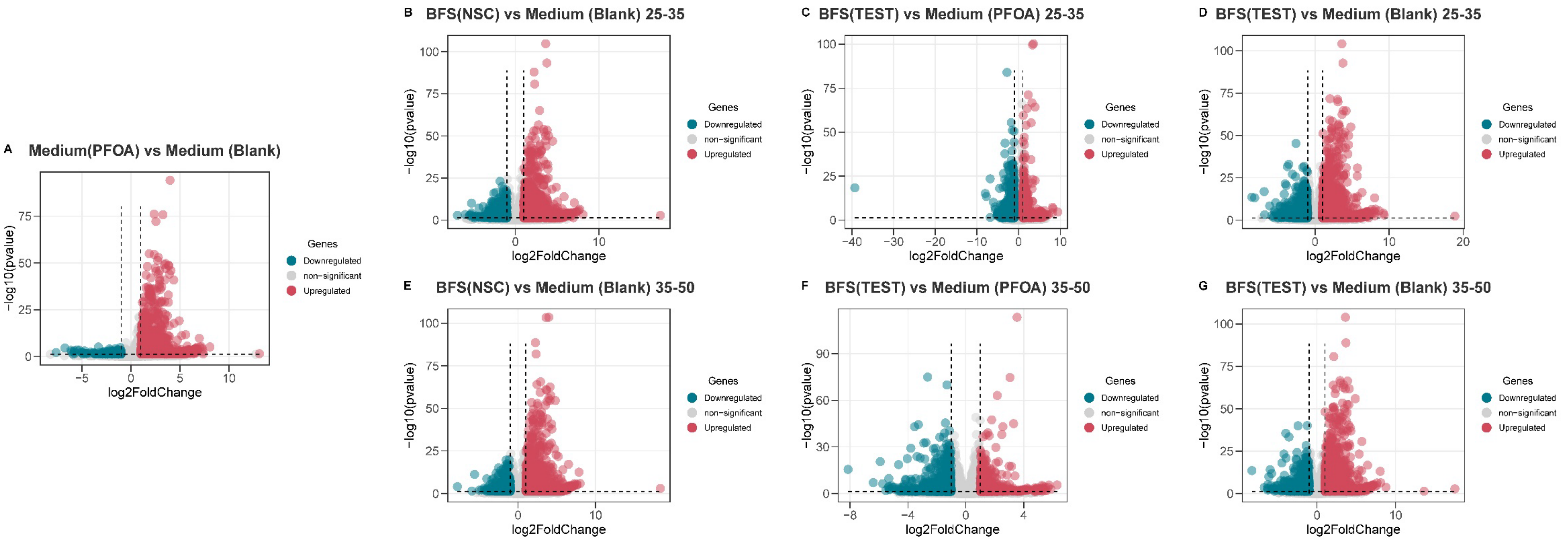

Figure S3 – Volcano plots showing differentially expressed genes.

	25-35 years			25 - 50 years			50 - 70 years		
**Phylum**	**Inoc**	**NSC**	**PFOA**	**Inoc**	**NSC**	**PFOA**	**Inoc**	**NSC**	**PFOA**
p__Actinomycetota	6.13E+07	1.86E+08	8.91E+07	9.94E+07	2.04E+08	1.23E+08	3.80E+07	1.45E+08	6.28E+07
p__Bacillota	4.62E+08	7.75E+08	4.80E+08	6.23E+08	9.07E+08	7.75E+08	6.05E+08	9.29E+08	7.35E+08
p__Bacteria_unclassified	6.76E+03		7.92E+03	4.90E+04					2.07E+03
p__Bacteroidota	2.54E+08	6.19E+08	5.48E+07	1.65E+08	5.48E+08	1.95E+07	1.90E+08	4.77E+08	4.85E+07
p__Candidatus_Melainabacteria	2.79E+05			5.36E+03			1.04E+06	3.33E+05	
p__Candidatus_Thermoplasmatota	1.25E+06	1.06E+07		1.36E+05	2.23E+06			3.48E+05	
p__Euryarchaeota	3.14E+06	1.26E+07	5.68E+06	1.41E+07	2.33E+07	1.70E+07	3.51E+06	1.61E+07	6.32E+06
p__Lentisphaerota	1.66E+05	3.67E+05			5.70E+05		2.78E+05	1.71E+06	
p__Mycoplasmatota	2.72E+04			3.41E+05			2.60E+06	4.11E+05	4.94E+04
p__Pseudomonadota	9.07E+06	8.15E+07	2.59E+08	8.84E+06	7.37E+07	1.70E+08	9.11E+06	4.73E+07	1.51E+08
p__Synergistota		1.47E+06		3.84E+05	3.50E+06	1.01E+05		5.25E+05	
p__Verrucomicrobiota	1.35E+07	3.78E+07	1.62E+07	1.05E+07	2.51E+07	7.71E+06	5.39E+06	1.52E+07	5.26E+06

Table S1 – Average phylum level abundances for each donor group. Cell colors scaled according to abundance, green is higher, red is lower.

EXTENDED MATERIALS AND METHODS FOR:

Perfluorooctanoic acid (PFOA) alters the structure of the gut microbial community and colonoid transcription

LinShu Liu[1*], Adrienne B. Narrowe[1], Jenni Firrman[1], Karley K. Mahalak[1], Venkateswari J. Chetty[1], Johanna M. S. Lemons[1], Aurélian Baudot[2], Pieter Van den Abbeele[2]

1 - Dairy and Functional Foods Research Unit, Eastern Regional Research Center, Agricultural Research Service, United States Department of Agriculture, Wyndmoor, PA 19038, USA

2 - Cryptobiotix SA., Ghent 9052, Belgium

*Corresponding Author: LinShu Liu, linshu.liu@usda.gov +1-215-233-6486

4.1. Materials

Perfluorooctanoic acid (PFOA) and all other chemicals were purchased from MiliporeSigma (Bornerm, Belgium), colonoids of distal colon tissue were kindly provided by Prof. G. Wu's Laboratory of Perelman School of Medicine, University of Pennsylvania (Philadelphia, Pennsylvania). Fecal samples donated by 18 adults at the ages belonging to three groups of 25-35, 35-50 and 50-70 years old (n = 6 for each) were collected according to the IRB protocol approved by the Ethics Committee of the University Hospital Ghent, Belgium (No. BC-09977). The selection criteria for donors were non-smokers, drank less than three servings of alcohol/day, had no gastrointestinal disorders or cancer, had a BMI < 30, were not on any medications to treat psychological disorders or allergies, and had no anti-/pre-/probiotics for at least three months prior to their donations.

4.2. *Ex vivo* fermentation

Using the *in vivo* SIFR® colonic stimulation, the interplay of PFOA and the gut microbiota was evaluated as described previously [1],[2]. Briefly, to each bioreactor containing anaerobic nutritional media, inoculated with fecal slurry from 3 age-groups, 25-35, 35-50, and 50-70 years old. For each age group, samples from 6 donors were tested with FPOA at the concentration of 10mg/L. The bioreactors were sealed; fermentations were carried out in anaerobic conditions at 37°C under constant magnetic stirring for 48

hours. Blank medium without PFOA was used as non-substance control (NSC), which was run in the same conditions for each age group in technical triplicates.

At the end of fermentation, a fraction of the culture was taken for bacterial cell counting; the remaining culture was centrifuged at 5,000g at 4°C for 10 min., the resultant pellet was stored at -80°C for DNA extraction and sequencing, the supernatant was filtered using 0.2μm filter to obtain bacterial free supernatant (BFS). All BFS obtained from PFOA included BFS(TEST) or NSC cultures BFS(NSC) were stored at -80°C for short chain fatty acid (SCFA) analysis and colonoid culture as described in following sections. For both the microbial composition and metabolite production analysis, each group was tested in six biological replicates. The test products were compared to NSC.

4.3. Metabolite measurement

Gas production was measured as the increase of pressure in the headspace of each vessel between the beginning and end of the experiment. Individual SCFA, acetate, propionate, butyrate, valerate, and branched short chain fatty acids (bSCFA) of isobutyrate, isovalerate and isocaproate) were determined as described previously [3]. Briefly, a fraction of each sample (0.5 mL) was mixed with three times (v/v) of distilled water, then dropwise acidified with 0.5 mL of 48% sulfuric acid, and 0.2 mL 2-methylhexanoic acid (internal standard) along with an excess of sodium chloride. The mixtures were homogenized by vertexing followed by standing on bench at room temperature for phase separation. The di-ethyl ether extracts were separated and subjected to SCFA analysis on a Trace 1300 chromatograph (Thermo Fisher Scientific, Merelbeke, Belgium). The instrument was equipped with a Stabilwax-DA capillary GC column, a flame ionization detector and a split injector, nitrogen gas was used as carrier phase. The analysis was implemented under following conditions: sample volume, 1.0 μL for each injection; starting temperature, 110 °C; end temperature, 240 °C; the temperatures of the injector and detector, 240 and 250 °C, respectively. Total SCFA and bSCFA amounts were calculated by summing the respective fatty acids. All measurements were triplicated.

4.4. Colonoid treatment and Transepithelial electrical resistance (TEER) measurement

Colonoids from distal colon tissue were seeded in 24-Well Hanging Inserts (cellQART, 0.4μm pore, polyethylene terephthalate membrane) at $1x10^5$ cells/well and cultured to form a confluent monolayer over a week. During that time colonoid cells were maintained in an incubator at 37°C with 5% CO_2, in 100μL colonoid growth media [4] in the apical chamber and 600μL in the basolateral chamber. Media was changed every 2-3 days. BFS(TEST) or BFS(NSC) from both 25-35 and 35-50 two age groups, six donors of each group, were diluted 1:4 in colonoid growth media (pH 7.2-7.4), filter-sterilized through a 0.2μm filter, and used to treat cells. Each diluted supernatant was used to treat one individual well. Experiments were repeated twice. Before treatment, plates of cells were removed from the incubator and allowed to equilibrate to room temperature for 20 minutes and then transepithelial electrical resistance (TEER) readings were measured with the EVOM2 epithelial voltohmmeter and STX4 electrode (World Precision Instruments) [5]. Media was aspirated and cells were rinsed once with PBS before 100μL of the diluted BFS was added to the apical chamber and 600μL of fresh growth media was added to the basolateral chamber. TEER was measured throughout a 24-hour period.

TEER readings for each treatment condition were averaged across donors for each age group. A separate experiment was performed to test whether PFOA in the absence of the bacterial metabolites affected barrier function. In this experiment, colonoid monolayers were treated in quadruplicate with 10mg/L PFOA Medium(PFOA) or DMSO (vehicle control) Medium(Blank) in colonoid growth media. TEER values were measured over 48 hours. Multiple comparisons of a 2-way ANOVA were performed in GraphPad Prism 10 (GraphPad Software, San Diego, CA) to determine significance at different time points.

4.5. Bacterial cell counts

Bacterial samples were suspended in anaerobic phosphate-buffered saline, stained with 1 μM SYTO 16, and counted for bacterial cell numbers [6] using a BD FACS Verse flow cytometer (BD, Erembodegem, Belgium)[7]. Data were analyzed using FlowJo, version 10.8.1.

4.6. DNA extraction and sequencing

DNA extraction and metagenomic sequencing was performed by SeqCenter (Pittsburgh, PA, USA). DNA extraction was performed using the ZymoBIOMICS DNA Miniprep Kit (Zymo Research, Irvine, CA, USA), and DNA concentration determined using a Qubit dsDNA assay (Thermo Fisher Scientific, Waltham, MA, USA.)

Illumina sequencing libraries were prepared using the tagmentation-based and PCR-based Illumina DNA Prep kit and custom IDT 10bp unique dual indices (UDI) with a target insert size of 280 bp. No additional DNA fragmentation or size selection steps were performed. Illumina sequencing was performed on an Illumina NovaSeq X Plus sequencer in one or more multiplexed shared-flow-cell runs, producing 2x151bp paired-end reads. Demultiplexing, quality control and adapter trimming was performed with bcl-convert1 (v4.2.4).

4.7. RNA extraction and sequencing

RNA extraction was performed using the QIAGEN RNeasy plus and QIAshredder kits (QIAGEN, Santa Clarita, CA, USA) with quantification using Qubit. RNA library preparation and sequencing was performed by Azenta Life Sciences (South Plainfield, NJ, USA.) The SMART-Seq v4 Ultra Low Input Kit for Sequencing was used for full-length cDNA synthesis and amplification (Clontech, Mountain View, CA), and the Illumina Nextera XT library preparation kit was used for sequencing library preparation (Illumina, San Diego, CA, USA.) Briefly, cDNA was fragmented, and adaptor was added using Transposase, followed by limited-cycle PCR to enrich and add index to the cDNA fragments. The sequencing library was validated on the Agilent TapeStation (Agilent Technologies, Palo Alto, CA, USA), and quantified by using a Qubit 3.0 Fluorometer (ThermoFisher Scientific, Waltham, MA, USA) as well as by quantitative PCR (KAPA Biosystems, Wilmington, MA, USA). Multiplexed sequencing libraries were sequenced on the Illumina NovaSeq using a 2x150bp Paired End (PE) configuration (Illumina, San Diego, CA, USA.) Image analysis and base calling were conducted by the NovaSeq Control Software (NCS). Raw sequence data (.bcl files) generated from Illumina NovaSeq was

converted into fastq files and de-multiplexed using Illumina bcl2fastq 2.20 software. One mismatch was allowed for index sequence identification.

4.8. Bioinformatics and statistical analysis

Raw shotgun metagenomic sequencing data was preprocessed using BBDuk v. 39.01 [8] to remove artifacts, contaminants and to perform read quality filtering and trimming. Trimmed, filtered reads were used as input to MetaPhlAn v. 4.1.1 with the mpa_vJun23_CHOCOPhlAnSGB_202403 reference database [9]. Individual sample profiles were combined to a single relative abundance table and phylum level taxonomy updated to reflect the NCBI taxonomy. Relative abundances were converted to absolute abundances using flow cytometry data for downstream analysis. Read-based functional profiling was performed using HUMAnN v. 4.0.0 [10]. Alpha diversity was calculated as taxonomic richness and the Shannon Diversity Index, using the MetaPhlAn utility script calculate_diversity.R, and tested for significant differences among treatments and age groups with Paired Wilcoxon signed-rank test. Beta diversity was calculated using weighted UniFrac distance and principal coordinate analysis (PCoA). PERMANOVA (pairwise adonis2) was used for testing significant clustering by treatment [11].

Testing for significant differences in taxon abundance with treatment was performed using MaAsLin3 [12] with the relative abundance table and flow cytometry cell counts for abundance normalization. Multiple testing correction was performed using the Benjamini-Hochberg method.

RNA-Seq data was quality trimmed using BBDuk as above. Transcript quantification was performed using kallisto v. 0.48.0 [13] using a reference prepared from the Ensembl human genome release 113 (Homo_sapiens.GRCh38.113.) Kallisto estimated abundances were used as input to DESeq2 v. 1.44.0 [14] to calculate differential gene expression using Wald's test. Lists of differentially expressed genes were used as input to enrichR [15] for GO bioprocess enrichment analysis. Other statistical analyses and visualizations used R/RStudio (v.4.1.3) using the packages: tidyverse (v.1.3.1) [16] , vegan (v.2.6-2) [17], ape (v.5.6-2) [18], ggvolc [19]. TEER plots were also created using GraphPad Prism 10 (GraphPad Software, San Diego, CA).

1. Lemons, J.M.S.; Narrowe, A.B.; Firrman, J.; Mahalak, K.K.; Liu, L.; Higgins, S.; Moustafa, A.M.; Baudot, A.; Deyaert, S.; Van den Abbeele, P. The food additive butylated hydroxyanisole minimally affects the human gut microbiome ex vivo. *Food Chem* **2025**, *473*, 143037, doi:10.1016/j.foodchem.2025.143037.
2. Firrman, J.; Deyaert, S.; Mahalak, K.K.; Liu, L.; Baudot, A.; Joossens, M.; Poppe, J.; Cameron, S.J.S.; Van den Abbeele, P. The Bifidogenic Effect of 2'Fucosyllactose Is Driven by Age-Specific Bifidobacterium Species, Demonstrating Age as an Important Factor for Gut Microbiome Targeted Precision Medicine. *Nutrients* **2024**, *17*, doi:10.3390/nu17010151.

3. Liu, L.e.a. Hydrogen Sulfide Has a Minor Impact on Human Gut Microbiota Across Age Groups. *Sci* **2025**, *7*, doi:10.3390/sci7030102.
4. VanDussen, K.L.; Sonnek, N.M.; Stappenbeck, T.S. L-WRN conditioned medium for gastrointestinal epithelial stem cell culture shows replicable batch-to-batch activity levels across multiple research teams. *Stem Cell Research* **2019**, *37*, doi:10.1016/j.scr.2019.101430.
5. Srinivasan, B.; Kolli, A.R.; Esch, M.B.; Abaci, H.E.; Shuler, M.L.; Hickman, J.J. TEER measurement techniques for in vitro barrier model systems. *J Lab Autom* **2015**, *20*, 107-126, doi:10.1177/2211068214561025.
6. Van den Abbeele, P.; Deyaert, S.; Thabuis, C.; Perreau, C.; Bajic, D.; Wintergerst, E.; Joossens, M.; Firrman, J.; Walsh, D.; Baudot, A. Bridging preclinical and clinical gut microbiota research using the ex vivo SIFR((R)) technology. *Front Microbiol* **2023**, *14*, 1131662, doi:10.3389/fmicb.2023.1131662.
7. Van den Abbeele, P.; Detzel, C.; Rose, A.; Deyaert, S.; Baudot, A.; Warner, C. Serum-Derived Bovine Immunoglobulin Stimulates SCFA Production by Specific Microbes in the Ex Vivo SIFR((R)) Technology. *Microorganisms* **2023**, *11*, doi:10.3390/microorganisms11030659.
8. Bushnell, B. *BBMap & BBTools*, 2015.
9. Blanco-Miguez, A.; Beghini, F.; Cumbo, F.; McIver, L.J.; Thompson, K.N.; Zolfo, M.; Manghi, P.; Dubois, L.; Huang, K.D.; Thomas, A.M.; et al. Extending and improving metagenomic taxonomic profiling with uncharacterized species using MetaPhlAn 4. *Nat Biotechnol* **2023**, *41*, 1633-1644, doi:10.1038/s41587-023-01688-w.
10. Beghini, F.; McIver, L.J.; Blanco-Miguez, A.; Dubois, L.; Asnicar, F.; Maharjan, S.; Mailyan, A.; Manghi, P.; Scholz, M.; Thomas, A.M.; et al. Integrating taxonomic, functional, and strain-level profiling of diverse microbial communities with bioBakery 3. *Elife* **2021**, *10*, doi:10.7554/eLife.65088.
11. Martinez Arbizu, P. pairwiseAdonis: Pairwise multilevel comparison using adonis. R package version 0.4. Available online: https://github.com/pmartinezarbizu/pairwiseAdonis (accessed on
12. Nickols, W.A.; Kuntz, T.; Shen, J.; Maharjan, S.; Mallick, H.; Franzosa, E.A.; Thompson, K.N.; Nearing, J.T.; Huttenhower, C. MaAsLin 3: Refining and extending generalized multivariable linear models for meta-omic association discovery. *bioRxiv* **2024**, doi:10.1101/2024.12.13.628459.
13. Bray, N.L.; Pimentel, H.; Melsted, P.; Pachter, L. Near-optimal probabilistic RNA-seq quantification. *Nature Biotechnology* **2016**, *34*, 525-527, doi:10.1038/nbt.3519.
14. Love, M.I.; Huber, W.; Anders, S. Moderated estimation of fold change and dispersion for RNA-seq data with DESeq2. *Genome Biol* **2014**, *15*, 550, doi:10.1186/s13059-014-0550-8.

15. Chen, E.Y.; Tan, C.M.; Kou, Y.; Duan, Q.; Wang, Z.; Meirelles, G.V.; Clark, N.R.; Ma'ayan, A. Enrichr: interactive and collaborative HTML5 gene list enrichment analysis tool. *BMC Bioinformatics* **2013**, *14*, 128, doi:10.1186/1471-2105-14-128.
16. Wickham, H.; Averick, M.; Bryan, J.; Chang, W.; D'Agostino McGowan, L.; François, R.; Grolemund, G.; Hayes, A.; Henry, L.; Hester, J.; et al. Welcome to the Tidyverse. *Journal of Open Source Software* **2019**, *4*, 1686, doi:https://doi.org/10.21105/joss.01686.
17. Oksanen, J. *Vegan: Community Ecology Package*, 2.6-6.1; 2024.
18. Paradis, E.; Schliep, K. ape 5.0: an environment for modern phylogenetics and evolutionary analyses in R. *Bioinformatics* **2019**, *35*, 526-528, doi:10.1093/bioinformatics/bty633.
19. Theodosiou, L. *ggvolc: Create volcano plots from RNA-seq data*, R package version 0.1.0; 2024.
